# Nosocomial and ventilator-associated pneumonia in a community hospital intensive care unit: a retrospective review and analysis

**DOI:** 10.1186/1756-0500-7-232

**Published:** 2014-04-11

**Authors:** Mehrdad Behnia, Sharon C Logan, Linda Fallen, Philip Catalano

**Affiliations:** 1 Georgia Health Sciences University, Doctors Hospital, Augusta, Georgia

**Keywords:** Pneumonia, Nosocomial, Ventilator-associated, Decortications, Empyema

## Abstract

**Background:**

Nosocomial and ventilator-associated pneumonia (VAP) are causes of significant morbidity and mortality in hospitalized patients. We analyzed a) the incidence and the outcome of pneumonias caused by different pathogens in the intensive care unit (ICU) of a medium-sized twenty-four bed community hospital and b) the incidence of complications of such pneumonias requiring surgical intervention such as thoracotomy and decortication.

**Results:**

We retrospectively reviewed the charts of patients diagnosed with nosocomial and ventilator-associated pneumonia in our ICU. Their bronchoalveolar lavage (BAL) and sputum cultures, antibiograms, and other clinical characteristics, including complications and need for tracheostomy, thoracotomy and decortication were studied. In a span of one year (2011–12), 43 patients were diagnosed with nosocomial pneumonia in our ICU. The median simplified acute physiology score (SAPS II) was 39. One or more gram negative organisms as the causative agents were present in 85% of microbiologic samples. The three most prevalent gram negatives were *Stenotrophomonas maltophilia* (34%), *Pseudomonas aeurginosa* (40%), and *Acinetobacter baumannii* (32%). Twenty eight percent of bronchoalveolar samples contained *Staphylococcus aureus*. Eight three percent of patients required mechanical ventilation postoperatively and 37% underwent tracheostony. Thirty five percent underwent thoracotomy and decortication because of further complications such as empyema and non-resolving parapneumonic effusions. *A. baumannii, Klebsiella pneumonia extended spectrum beta lactam (ESBL) and P. aeurginosa* had the highest prevalence of multi drug resistance (MDR). Fifteen patients required surgical intervention. Mortality from pneumonia was 37% and from surgery was 2%.

**Conclusion:**

Nosocomial pneumonias, in particular the ones that were caused by gram negative drug resistant organisms and their ensuing complications which required thoracotomy and decortication, were the cause of significant morbidity in our intensive care unit. Preventative and more intensive and novel infection control interventions in reducing the incidence of nosocomial pneumonias are strongly emphasized.

## Background

Nosocomial pneumonias, including ventilator-associated, are the leading cause of infections in a hospital setting. Their economic burden is financially significant, resulting in considerable resource utilization in an era of global financial constraint [1]. Mean cost of hospitalization and treatment could be as high as 100,000 dollars for a case of VAP. Therefore, prevention of pneumonia in a hospital setting is now considered a formidable challenge. This issue becomes more important when pathogen resistance is on the rise and available novel antimicrobial agents are scarce [1].

Aside from economic constraints, nosocomial pneumonias could be potentially fatal because of emergence of resistance pathogens. Incidence of gram negative organisms such as *P. aureoginosa, S. multophilia*, and *K. pneumoniae* in addition to methicillin resistant *Saphylococcus aureus* (MRSA) are on the rise [2,3]. The numbers are alarming especially when the resistance to potent antimicrobials such as quinolones and carbapenems is escalating [4,5].

We retrospectively studied the incidence of nosocomial pneumonia, including VAP, in our hospital intensive care unit, which is a medium-sized community hospital, for a period of one year. Clinical characteristics including length of stay, antibiotic sensitivity and resistance antibiograms of the offending organisms were studied in detail. We further studied complications caused by pneumonia requiring surgical interventions of tracheostomy, thoracotomy, and decortications. Final outcome and mortality data are also included. We try to convey the message that thoracotomy and decortication that are historically considered to be interventions of high morbidity and mortality, could be done safely with low mortality in an era of multidrug resistant complicated pneumonias by an experienced team in a medium sized hospital.

## Methods

### Diagnosis of pneumonia

The following criteria were used when a diagnosis of pneumonia in ICU was made: radiographic appearance of a new infiltrate or consolidation, temperature of greater than 100.5 F, leukocytosis, and increase in tracheobronchial mucus production; other criteria which may have been present included worsening oxygenation and positive blood culture [6]. The ICU was a 24-bed, with an annual admission of 1420 patients.

### Microbiology

Microbiologic diagnosis was made by obtaining sample of tracheobroncial tree by endotracheal suctioning or by bronchoalveolar lavage through bronchoscopy [6]. Quantitative culture analysis was done in microbiology laboratory which is as follows: in brief bronchoalveolar lavage fluid was incubated aerobically at 35 degrees or under 5% CO_2_ per published laboratory protocols. The plates were read for growth after 24 hours of incubation and the pathogen was worked up accordingly. Another reincubation was done for 24 hours and plates were read for growth the following day. Recovery of more than 100,000 colony forming unit (CFU)/ml in a lavage sample was considered to be positive.

Culture positivity was based on the number of culture colony-forming units greater than or equal to 10^4^/mm as the threshold value for a positive culture. Bacterial identification and antibiotic susceptibility testing with minimum inhibitory concentration (MIC) panel was performed as per existing laboratory protocols.

### Thoracotomy

Open decortication through an anterior approach involving limited thoracotomy was performed by one cardiothoracic surgeon for all the patients requiring surgery. Decision for surgery was made if there was presence of a non-resolving, organizing and persistent pleural effusion which was refractory to conventional treatments including antimicrobial therapy and chest tube placement; other criteria for surgery included lack of improvement in patient’s condition, worsening of oxygenation, fever, and leukocytosis. Computerized tomography (CT) scan of chest was done in all patients requiring surgery. We did not use streptokinase or any fibrinolytic agent through chest tube instillation in any of our patients prior to surgery.

### Other criteria

Other comorbidities were calculated in every patient using SAPS II score. Patients, who met criteria for end organ dysfunction and septic shock, received activated protein C or drotrecogin alfa, per existing protocol at our institution as follows:

Patient had to meet at least 3 of the following: core temperature greater than 38C or less than 36C; heart rate greater than 90 beats/minute, respiratory rate greater than 20/minute or PaCO_2_ less than 32 mmHg or the use of mechanical ventilation for an acute respiratory process, WBC greater than 12,000/mm^3^ or less than 4000/mm3 or a differential count with > 10% immature neutrophils; furthermore patients had to meet at least one of the following: a) arterial systolic blood pressure < 90 mmHg or mean arterial BP < 70 mmHg for at least 1 hour despite fluid resuscitation or the use of vasopressor in an attempt to maintain a systolic BP > 90 mmHg, b) urine output < 0.5 ml/kg for one hour despite adequate fluid resuscitation, c) PaO_2_ to FIO_2_ ratio < 250 in the presence of other dysfunctional organs/systems or < 200 if the lung was only dysfunctional organ; d) platelet count < 80,000/mm^3^ or a decrease by 50% in the 3 days prior to enrollment; e) unexplained acidosis with a pH < 7.3 or the base deficit of > 5 mmol/L in association with a plasma lactate level that was > 1.5 times the upper limit of normal.

### Statistical analysis

Data were incorporated into a spreadsheet and subsequently imported into the statistical program systat. Descriptive statistics were assessed and chi square tests were used to determine relationships between variables (e.g., between organism and death). Incidence was assessed by the number of subjects in a given category relative to the total population.

## Results

**Figure 1:**
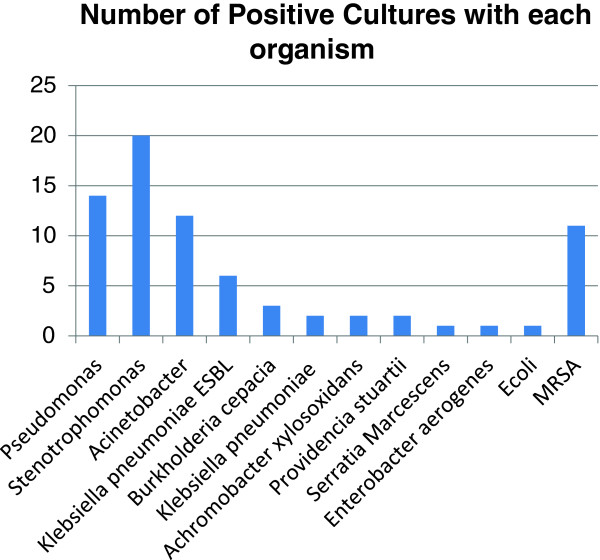
Bar graph depicting the frequency of microorganisms as causative agents of pneumonia.

**Figure 2:**
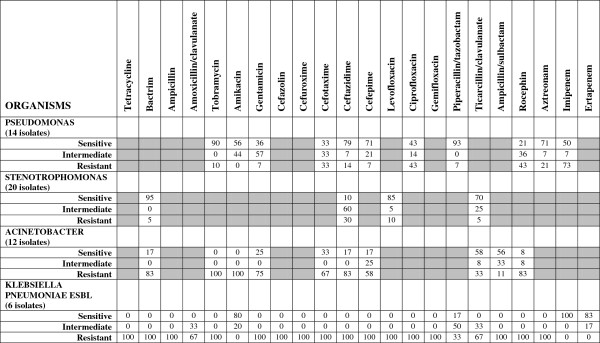
**Isolated microorganisms of the respiratory tract in patients with nosocomial and ventilator-associated pneumonia and their *in vitro *** **antibiotic sensitivity (total number of isolates=75).**

**Figure 3:**
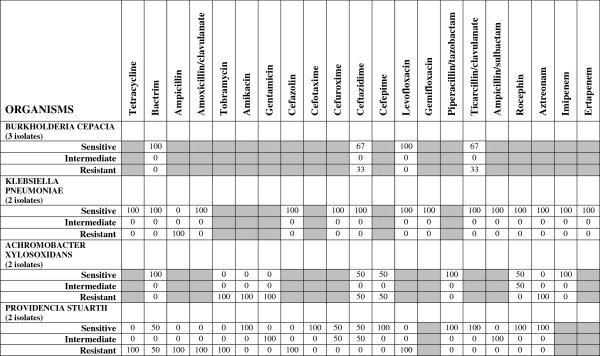
**Isolated microorganisms of the respiratory tract in patients with nosocomial and ventilator-associated pneumonia and their *in vitro *** **antibiotic sensitivity (total number of isolates=75).**

**Figure 4:**
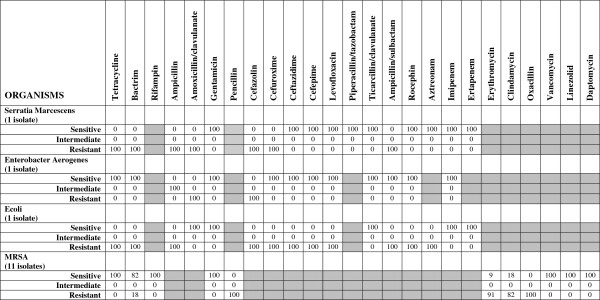
**Isolated microorganisms of the respiratory tract in patients with nosocomial and ventilator-associated pneumonia and their *in vitro *** **antibiotic sensitivity (total number of isolates=75).**

Forty three patients met the clinical and microbiologic criteria for diagnosis of nosocomial pneumonia. Some had more than one organism on microbiology culture of endobronchial tree. A total of 75 cultures for different organisms were positive (Tables 1, 2, 3 and 4). Of the 43 patients with nosocomial pneumonia, 36 were on mechanical ventilation. Two had prior tracheostomy. Sixteen of 43 required tracheostomy during their ICU stay because of inability to be weaned off the ventilator. Fifteen of the 43 patients (35%) developed non-resolving complicated parapneumonic effusion with or without empyema and required to undergo thoracotomy and decortications. All the surgeries were performed by one cardiothoracic surgeon. Twenty three of the 43 patients had diagnosis of septic shock requiring vasopressor support. Summary of findings and other comorbidities are detailed in Tables 1, 2, 3, 4, and 5. Four patients met criteria for initiation of drotrecogin alfa. Sixteen of the 43 patients died (37%). The incidence of organisms and their antibiotic sensitivity and resistance are shown in Figures 1, 2, 3 and 4. Nurse to patient ratio was 1 to 2 for all of the patients.

**Table 1:** Demographic and outcome of patients with nosocomial and ventilator associated pneumonia

**Study population and clinical summary**	
**N**	43 patients
**Gender (male/female)**	18/25
**Age (yrs)**	65.9 ± 16.8 (range 26-93)
**Length of hospital stay (days)**	26.7 ± 15.9 (range 5-71)
**Average day of hospital stay patient developed positive cultures for noscomial pneumonia**	9.6 ± 6.4 (range 2-28)
**Discharge plan**	Home: 6 patients
	Other Facility: 21 patients
	Death: 16 patients

**Table 2:** Mortality and its association with microbiology of cause of pneumonia

**Microbiologic agent cause of mortality**	**Number of death (Nosocomial; VAP; Total)-percent of total**
*Pseudomonas*	(3;6;9)- 21%
*MRSA*	(2;2;4)- 9%
*Stenotrophomonas, Acinetobacter, or both*	(7;4;11)- 26%
*Klebsiella (ESBL)*	(0;1;1)- 2%
*Other agents(none of the above)*	(4;0;4)- 17%
*Multidrug Resistance*	(7;3;10)- 23%
*Gram stain*	Gram positive: (9;1;10)-23%
	Gram negative: (9;7;16)-37%

**Table 3:** Characteristics of patients with mortality

**Characteristics**	**Number of death (percent of total)**
Renal failure requiring dialysis	3 (7%)
Received Drotrecogin alfa	2 (4.6%)
Presentation of septic shock	10 (23%)
Postoperative bleeding requiring surgical intervention	1 (2%)
Postoperative Myocardial infarction	3 (9%)

**Table 4:** Interventions and complications in patients with nosocomial and ventilator associated pneumonia

**Need for mechanical ventilation:**	36 patients
**Need for tracheostomy during hospitalization:**	16 patients
**Chronic tracheostomy prior to admission:**	2 patients
**Requirement for surgery including chest tube, thoracotomy/decortication:**	15 patients
**Stenotrophomomas**	7
**Acinetobacter**	3
**Pseudomonas**	8
**MRSA**	3
**Others**	4
**Septic shock:**	23 patients
**Required drotrecogin alfa:**	4 patients
**Average SAPS II score:**	39

**Table 5:** Variables and their assigned severity score used in calculation of Simplified Acute Physiologic Score II (SAPS II)

**Variable**	**Range**	**Points**
Patient age	< 40 years	0
	40-59 years	7
	60-69 years	12
	70-74 years	15
	75-79 years	16
	> 80 years	18
Type of admission	Scheduled surgery	0
	Medical	6
	Unscheduled surgery	8
Temperature	< 39° C, < 102.2° F	0
	≥ 39° C, ≥ 102.2° F	3
Systolic blood pressure	≥ 200 mmHg	2
	100-199 mmHg	0
	70-99 mmHg	5
	< 70 mmHg	13
Heart rate	≥ 160 bpm	7
	120-159 bpm	4
	70-119 bpm	0
	40-69 bpm	2
	< 40 bpm	11
Glasgow coma scale	14-15	0
	11-13	5
	9-10	7
	6-8	13
	< 6	26
Urine output	≥ 1 L/24 hours	0
	0.5-0.999 L/24 hours	4
	< 0.5 L/24 hours	11
White blood cell count	< 1000/ mm^3^	12
	1000-19,000/mm^3^	0
	≥ 20,000/mm^3^	3
Blood urea nitrogen	≥ 30 mmol/L, ≥ 84 mg/dL	10
	10-29.9 mmol/L, 28-83 mg/dL	6
	< 10 mmol/L, < 28 mg/dL	0
Potassium level	< 3 mEq/L	3
	3-4.9 mEq/L	0
	≥ 5mEq/L	3
Sodium level	< 125 mEq/L	5
	125-144 mEq/L	0
	≥ 145 mEq/L	1
Bicarbonate level	< 15 mEq/L	6
	15-19 mEq/L	3
	≥ 20 mEq/L	0
Bilirubin level	< 4 mg/dL, < 68.4 micromol/L	0
	4-5.9 mg/dL, 68.4-102.5 micormol/L	4
	≥ 6 mg/dL, ≥ 102.6 micromol/L	9
PaO2/FiO2 (if mechanically ventilated or receiving CPAP)	< 100 mmHg	11
	100-199 mmHg	9
	≥ 200 mmHg	6
AIDS	Yes	17
	No	0
Metastatic carcinoma	Yes	9
	No	0
Hematologic malignancy	Yes	10
	No	0

There were no strong relationships between multi drug resistant strains and death or surgical intervention, between VAP and nosocomial pneumonia or between gram (−) and gram (+). However the association between MDRS and death had a chi square statistic of 0.13 which was suggestive of a relationship. In addition, there were borderline relationships between other organisms and death including *Stenotrophomonas*/*Acinetobacter*/or Both (chi square statistic 0.11), *Klebsiella* (ESBL) (0.16), Other agents (none of above) (0.06) and if subjects were Gram Positive (0.12).

## Discussion

Our data provide the following results: a) Gram negatives and MRSA are leading causes of nosocomial and VAP in our intensive care; b) previously uncommon gram negatives such as *Stenotrophomonas maltophilia* and *Acinetobacter baumannii* are becoming more prevalent; c) a large group of patients further required thoracotomy, decortication, and tracheostomy because of ensuing complication; d) thoracotomy and decortication can be done safely in patients with complicated and non resolving chest infections and pneumonias with a low mortality.

Nosocomial pneumonia is a disease with significant morbidity, mortality, and economic burden. Prevention plays a considerable role in reducing the incidence of this disease. Use of VAP prevention bundles appears to lower its incidence. Despite implementation of strict preventative bundle (subglottic suctioning, head of the bed elevation, strict hand washing, use of rotational specialty beds) the incidence of VAP remains high.

At our institution, a high incidence of gram negative organisms, in particular uncommon pathogens such as *A. baumannii* and *S. maltophilia,* has been of concern. Their incidence is surpassing other common gram negative organisms such as *P. aeruginosa*, and *K. pneumoniae*. Their high incidence also surpasses MRSA (Table 4). The trend is of concern and the exact cause has yet to be clearly established. One previous report from our institution identified the organisms as the cause of a pseudo outbreak [7]; the cause was traced back to a contaminated bronchoscope; however we doubt that this is the cause for the high number of these organisms as the cause of pneumonias because of a very strict infection control policy that has been implemented since the outbreak occurred. This includes interventions such as use of dedicated bronchoscopes for the intensive care unit and use of disposable and sterile drapes.

One possible reason for the high trend of infections is because our center is a tertiary referral center for the outlying smaller medical centers, with a high admission rate for complex medical problems. The other reason may be because of our large Burn Center which is the largest in the Southwest United States, although the statistics of our burn unit is not included in our study.

Patients affected with pneumonias caused by *A. baumannii* and *MRSA* have been shown to be mechanical ventilator-dependent for a longer time and to cause more bacteremia [8]. Also SAPS II score was an independent variable associated with a higher mortality. In one study the average SAPS II score was 51.5 for pneumonia patients with bacteremia and 46.6 for pneumonia patients without bacteremia [8]. In the same study, septic shock was seen in approximately 38% of patients in both groups and mortality of bacteremic and non-bacteremic patients were 57% and 33%, respectively. Interestingly, the same study which was a large multicenter, multinational, cohort, prospective study showed that *MRSA* was the isolated organism causing pneumonia in 14% of patients. In the same study, *Pseudomonas* species was the organism isolated in 17%, *A. baumannii* in 14%, and *S. maltophilia* in 2.6% of respiratory tract isolated cultures (Figure 1).

We did not study the rate of bacteremia caused by each specific organism; however, previous studies have shown that *A. baumannii* and *MRSA* are independent predictors of bacteremia after confounders have been adjusted [8]. The conclusion drawn in another cohort matched study by Fagon et al. demonstrates that pneumonias in ventilated patients with *Pseudomonas* or *Acinetobacter* species cause considerable mortality as high as 71% [9]; the subject is controversial and been debated intensively; there are other studies that have shown that *Acinetobacter*-caused VAP is not associated with either significant mortality or increased length of stay [10].

In a previous study *Acinetobacter* pneumonia was reported to be about 6% of VAPs; in our study, this organism was the causative agent in 15% of patients with nosocomial pneumonia. We do not know why our institutional numbers were high; previously we reported a pseudo outbreak caused by a contaminated bronchoscope [7]; one possible source could be presence of a large ICU for Burn victims at our institution as the source of cross contamination. Other likely risk factors include prior sepsis and antibiotic use, reintubation, duration of hospital stay and mechanical ventilation, and the use of imipenem and fluoroquinolone antibiotics [11].

*Pseudomonas* pneumonia resistance to imipenem has been reported in some studies to be around 20 percent [12].This is very comparable to our present statistics. The trend may be related to over use of imipenems as a first line therapy in treatment of *Pseudomonas* and reluctance of clinicians to de-escalate coverage if not needed [12].

*Stenotrophomonas maltophilia*, in a prospective observational case–control study of patients in a 30-bed ICU, was shown to cause only 2% of pulmonary tract colonizations and infections with this gram negative organism. Chronic obstructive pulmonary disease (COPD) and duration of antibiotic treatment were independent risk factors. Mortality rates, duration of mechanical ventilation and of ICU stay, were significantly elevated in this infection compared to controls [13]. Our institutional positive culture rate (20 of 75) was significantly higher compared to the above study. The exact reason behind this is unknown but may be related to a higher SAPS II score of our patients compared to the above study (39 vs. 31). Nevertheless, this discrepancy warrants further investigation.

Nowadays, MRSA accounts for 20%–40% of all hospital-acquired and ventilator-associated pneumonias [14]. All of our institutional strains were vancomycin and linezolid sensitive. Important factors in prevention of MRSA pneumonia is strict infection control implementation and application of VAP bundles. These include elevation of head of the bed, daily sedation break and assessment for extubation, peptic ulcer prophylaxis, use of endotracheal tubes with subglottic suctioning [15], and oropharyngeal decontamination [15].

An interesting finding of our data was the high number of patients requiring tracheostomy and thoracotomy and decortications in our cohort. Thoracotomy was used mainly in complicated and non resolving parapneumonic effusions and empyemas as sequels of serious pneumonias. The outcome data of our center with thoracotomy has been excellent since decortication for entrapped lung could be a serious and complicated surgery with high mortality [16]. However, at our center the mortality following decortication through open thoracotomy or video-assistance was only 2 percent. This is a remarkably good statistics. We speculate the good outcome to be related to intensive and rigorous postoperative care, competency of one dedicated surgeon performing a high volume of surgeries, and diligent postoperative nursing care. Our hospital had the highest number of decortication surgeries in comparison to other hospitals in the state of Georgia (personal communication).

Thoracotomy and decortication as surgical interventions for treatment of empyema and entrapped lung is being seriously debated and the final verdict is not announced conclusively as of yet. There is no good randomized blinded controlled study that has compared this extensive surgery with traditional interventions such as chest tube placement and other interventions such as debridement via video-assisted thoracoscopic surgery (VATS) and open window thoracotomy [17]. The major confounding factors in studies that have been published include, but are not limited to, age and degree of organization of infected/parapneumonic effusion, severity of illness in the patient (as measured by SAPS II score), age of patient, and experience of the surgeon [17]. It appears that in most studies the results are significantly different based on the institution and most studies provide level 2b or lower level recommendations [17]. The experience and competence of the surgeon, quality of care by the nursing staff and the intensivist physician, and patients’ co morbidities are significant factors in the final outcome. There are also other studies such as the observational retrospective study by Kho et al. which showed that debridement alone without decortication in patients with empyema eventually caused the same re-expansion of pleural cavity space after evacuation that was not significantly different from empyema decortication [18]. It seems like that there are more surgeons who refrain from open thoracotomy and decortications and are instead favoring other interventions such as VATS for management of pleural effusions complicating pneumonias; VATS is now being favored by experienced surgeons in academic and also tertiary referral centers as a procedure with lower 30-day mortality, sepsis and other postoperative complications in comparison to open thoracotomy and decortications [19].

Our center has a very robust and exceptional experience with decortications for complicated parapneumonic effusions, entrapped lungs, and empyemas as complications of nosocomial pneumonias. We use a limited anterior thoracotomy which significantly lowers the morbidity of surgery with lesser effects on lung mechanics. Our mortality, postoperative septicemia, prolonged chest tube air leak, and tracheostomy appear to be lower compared to other published studies, although our outcomes have not been published officially yet. Our results appear to be exceptionally remarkable for a medium sized community hospital since high volume of open decortications appears to be a feature of academic institutions and large tertiary referral medical centers which have the largest published experience with this type of surgery. We did not use streptokinase or any other fibrinolytic agent in the chest tubes. Our previous unpublished experience has shown that fibrinolytic instillation did not result in any significant improvement of outcome. Our surgical team prefers limited or extensive thoracotomy to fibrinolytic therapy.

Sufficient nursing staffing to provide high quality ICU care is a very crucial factor in reduction of nosocomial pneumonias which is regrettably often ignored and not appreciated by other health care providers [15]. In our ICU, the nurse to patient ratio is established at 1:2 and for high acuity patients, e.g. around-the-clock dialysis, is 1:1. This practice has had significant impact on quality of care and therefore a positive impact in reduction of the incidence of nosocomial pneumonia. This practice in conjunction with strict infection control measures, e.g., hand washing, quarantine of MRSA colonized patients, wearing of disposable gown and glove in quarantined rooms, has shown to be very helpful in lowering cross contamination between patients and therefore reduction of nosocomial infections.

The limitations of our study were several. It was a retrospective study. Selection of antibiotics for treatment of pneumonia was physician-dependent and there was not a selection protocol that had been present and therefore each physician might have used a different choice of antibiotic for treatment of a specific organism. The same limitation held true for recommending thoracotomy and decortication. There was only one surgeon that performed all the surgeries; however, decision for consulting the surgeon and requesting surgery was dependent on the attending intensivist or non-intesivist physician. Another limitation of our study was that the study was not done in a “closed” ICU, meaning that there was not a dedicated intensivist who would assume full medical care of all the patients. There were other non-intensivists that assumed patient’s care and the decision making process, was not similar amongst physicians.

## Conclusion

The 4 most common organisms causing nosocomial and VA pneumonias at our intensive care unit were *Stenotrophomonas maltophilia*, *Pseudomonas aeurginosa*, *Acinetobacter baumannii*, and MRSA. *A. baumannii, Klebsiella pneumonia, and P. aeurginosa* were the most frequent organisms that had multi drug resistance. Thoracotomy and decortication are life saving procedures with very good outcomes that could be done safely with a very low morbidity and mortality by an experienced surgeon.

## Competing interest

The authors declare that they have no competing interest.

## Authors’ contributions

MB: participated in format, design, and authorship of the manuscript; SL: was in charge of data collection and draft of the tables; LF: coordinated the data collection; PC: the cardiothoracic surgeon who performed all the surgeries; all authors read and approved the final manuscript. “This research, as undertaken, was not designed as a clinical investigation (as defined by U.S. 21CFR56.102(c)) of a drug, device or biologic. At the time of data analysis, the research was exempt from IRB oversight consistent with U.S. federal regulations at 45CFR46.101(b) (4) (“research involving the collection or study of existing data, documents, records, pathological specimens, or diagnostic specimens, if these sources are publicly available or if the information is recorded by the investigator in such a manner that subjects cannot be identified, directly or through identifiers linked to the subjects”). The retrospective data collection was performed and analyzed anonymously and therefore written informed consent was not required”.

## References

[B1] Kollef MH Vancomycin for methicillin-resistant Staphylococcus aureus pneumonia: the good, the bad, and the ugly Crit Care Med 2012 40 1 330 2 10.1097/CCM.0b013e31822e5741 22179367

[B2] Micek ST Welch EC Khan J Pervez M Doherty JA Reichley RM Hoppe-Bauer J Dunne WM Kollef MH Resistance to empiric antimicrobial treatment predicts outcome in severe sepsis associated with Gram-negative bacteremia J Hosp Med 2011 6 7 405 10 10.1002/jhm.899 21916003

[B3] Venier AG Gruson D Lavigne T Jarno P L'Heriteau F Coignard B Savey A Rogues AM Identifying new risk factors for Pseudomonas aeruginosa pneumonia in intensive care units: experience of the French national surveillance, REA-RAISIN J Hosp Infect 2011 79 1 44 8 10.1016/j.jhin.2011.05.007 21741117

[B4] Apisarnthanarak A Warren DK Fraser VJ The long-term outcome of a multifaceted intervention to reduce ventilator-associated pneumonia: can zero really be achieved? Am J Infect Control 2011 39 7 613 4 10.1016/j.ajic.2010.11.010 21641086

[B5] Celik IH Oguz SS Demirel G Erdeve O Dilmen U Outcome of ventilator-associated pneumonia due to multidrug-resistant Acinetobacter baumannii and Pseudomonas aeruginosa treated with aerosolized colistin in neonates: a retrospective chart review Eur J Pediatr 2012 171 2 311 6 10.1007/s00431-011-1537-z 21809011

[B6] American Thoracic Society and Infectious Diseases Society of America Guidelines for the management of adults with hospital-acquired, ventilator-associated, and healthcare-associated pneumonia Am J Respir Crit Care Med 2005 171 4 388 416 15699079 10.1164/rccm.200405-644ST

[B7] Behnia M Amurao K Clemons V Lantz G Pseudo-outbreak of Stenotrophomonas maltophilia and Acinetobacter baumannii by a contaminated bronchoscope in an intensive care unit Tanaffos 2010 9 3 44 9

[B8] Magret M Lisboa T Martin-Loeches I Manez R Nauwynck M Wrigge H Cardellino S Díaz E Koulenti D Rello J EU-VAP/CAP Study Group Bacteremia is an independent risk factor for mortality in nosocomial pneumonia: a prospective and observational multicenter study Crit Care 2011 15 1 R62 10.1186/cc10036 21324159 PMC3221995

[B9] Fagon JY Chastre J Hance AJ Montravers P Novara A Gibert C Nosocomial pneumonia in ventilated patients: a cohort study evaluating attributable mortality and hospital stay Am J Med 1993 94 3 281 8 10.1016/0002-9343(93)90060-3 8452152

[B10] Garnacho J Sole-Violan J Sa-Borges M Diaz E Rello J Clinical impact of pneumonia caused by Acinetobacter baumannii in intubated patients: a matched cohort study Crit Care Med 2003 31 10 2478 82 10.1097/01.CCM.0000089936.09573.F3 14530754

[B11] Hartzell JD Kim AS Kortepeter MG Moran KA Acinetobacter pneumonia: a review MedGenMed 2007 9 3 4 18092011 PMC2100077

[B12] Zilberberg MD Chen J Mody SH Ramsey AM Shorr AF Imipenem resistance of Pseudomonas in pneumonia: a systematic literature review BMC Pulm Med 2010 10 45 10.1186/1471-2466-10-45 20796312 PMC2939581

[B13] Nseir S Di Pompeo C Brisson H Dewavrin F Tissier S Diarra M Boulo M Durocher A Intensive care unit-acquired Stenotrophomonas maltophilia: incidence, risk factors, and outcome Crit Care 2006 10 5 R143 10.1186/cc5063 17026755 PMC1751051

[B14] Rubinstein E Kollef MH Nathwani D Pneumonia caused by methicillin-resistant Staphylococcus aureus Clin Infect Dis 2008 46 Suppl 5 S378 85 18462093 10.1086/533594

[B15] Craven DE Preventing ventilator-associated pneumonia in adults: sowing seeds of change Chest 2006 130 1 251 60 10.1378/chest.130.1.251 16840410

[B16] Colice GL Curtis A Deslauriers J Heffner J Light R Littenberg B Sahn S Weinstein RA Yusen RD Medical and surgical treatment of parapneumonic effusions : an evidence-based guideline Chest 2000 118 4 1158 71 10.1378/chest.118.4.1158 11035692

[B17] Molnar TF Current surgical treatment of thoracic empyema in adults Eur J Cardiothorac Surg 2007 32 3 422 30 10.1016/j.ejcts.2007.05.028 17646107

[B18] Kho P Karunanantham J Leung M Lim E Debridement alone without decortication can achieve lung re-expansion in patients with empyema: an observational study Interact Cardiovasc Thorac Surg 2011 12 5 724 7 21345817 10.1510/icvts.2010.247619

[B19] Tong BC Hanna J Toloza EM Onaitis MW D'Amico TA Harpole DH Burfeind WR Outcomes of video-assisted thoracoscopic decortication Ann Thorac Surg 2010 89 1 220 5 10.1016/j.athoracsur.2009.09.021 20103240

